# 4425 Anibiotic-Resistant Organism Acquisition in Nursing Facility Patients

**DOI:** 10.1017/cts.2020.54

**Published:** 2020-07-29

**Authors:** Joyce Wang, Marco Cassone, Kristen Gibson, Bonnie Lansing, Lona Mody, Evan Snitkin, Krishna Rao

**Affiliations:** 1 University of Michigan School of Medicine; 2 University of Michigan

## Abstract

OBJECTIVES/GOALS: We investigated the association between gut microbiota features in newly admitted nursing facility (NF) patients and the acquisition of vancomycin-resistant *Enterococcus* (VRE) and/or resistant Gram-negative bacteria (rGNB) within 14 days. METHODS/STUDY POPULATION: Patients were recruited at 6 Michigan NFs from 09/16-08/18. VRE or rGNB colonization status was determined by culture swabs collected from multiple body sites at enrolment, day 7, and day 14. Our analysis focused on patients with no colonization at baseline, a perirectal swab collected at baseline, and at least one follow-up visit. The V4 hypervariable region of the 16S rRNA gene from bacterial DNA in each sample was PCR-amplified and sequenced on the MiSeq platform. Sequencing results were then processed with the *mothur* bioinformatics pipeline to classify bacterial taxa present in each sample. Taxa typically associated with the skin microbiota were removed. The primary outcome was acquisition of VRE and/or rGNB within 14 days. Exposures of interest included patient and microbiota characteristics. RESULTS/ANTICIPATED RESULTS: Among 61 patients, 18 (30%) acquired AROs within 14 days of enrolment (3 VRE, 13 rGNB, 2 both) (**Table 1**). The baseline microbiota features differed significantly in those who acquired a new ARO. Of the major 8 phyla found across samples, patients who acquired an ARO were depleted in the number of phyla present (5.74 ± 1.20 vs 5.06 ± 1.43; p = 0.037) (**Fig. 1**). The log10-transformed relative abundance of *Enterococcus* was enriched in patients who acquired an ARO (−0.32 ± 1.47) compared to those who did not (−1.68 ± 1.76; p = 0.021) (**Fig. 2**). Patients who did not acquire an ARO tended to harbour more butyrate-producing bacterial taxa and strict anaerobes, although the differences were not statistically significant (relative abundance of butyrate producer: 29.49 ± 22.09 vs 22.05 ± 17.76; anaerobes: 64.78 ± 23.54 vs 53.68 ± 27.61). DISCUSSION/SIGNIFICANCE OF IMPACT: Microbiota metrics calculated from perirectal samples are predictive of ARO acquisition. The clinical utility of perirectal samples thus warrants further assessment.

